# Parental and training coaches’ knowledge and attitude towards dental trauma management of children

**DOI:** 10.1111/adj.12913

**Published:** 2022-05-17

**Authors:** J Tian, JJJ Lim, FKC Moh, A Siddiqi, J Zachar, S Zafar

**Affiliations:** ^1^ School of Dentistry The University of Queensland Brisbane Queensland Australia; ^2^ School of Dentistry and Oral Health Griffith University Southport Queensland Australia

**Keywords:** Children, coach, knowledge, parent, trauma

## Abstract

**Background:**

The aim of this study was to evaluate parental and training coaches’ knowledge and attitude towards traumatic dental injuries (TDIs) among children.

**Material and Methods:**

A 31‐item questionnaire was distributed to the parents and training coaches attending local sporting clubs in Brisbane region, Australia. The questionnaire consisted of five parts (i) demographic and professional information; (ii) TDIs in the primary dentition; (iii) fractures and subluxation of permanent teeth (iv) avulsion of permanent teeth, and (v) information and knowledge related to the management of traumatized teeth. The jamovi (Version 1.6.3) and GraphPad Prism were used for data analysis.

**Results:**

A total of 233 participants were surveyed, 211 parents and 22 coaches. Of all types of injuries, parental knowledge of managing avulsion to permanent teeth was poorest (9.5%), followed by management of injuries of primary teeth (17.5%) and management of fractures or subluxation of permanent teeth (29.4%). Parents in health care occupations had higher satisfaction on self‐knowledge in managing TDIs however there was no significant difference in knowledge levels between health care personnel and other professions (*P* = 0.128). There was a discrepancy between the lack of knowledge and willingness to further self‐educate with online platforms being the preferred medium.

**Conclusion:**

The study showed a gap in parents’ and training coaches’ knowledge regarding the management of TDIs among children.

**Abbreviations and acronyms**
DTG: dental trauma guideline
IADT: International Association of Dental Traumatology
PDL: periodontal ligament
TDI: traumatic dental injury

## INTRODUCTION

Traumatic dental injuries (TDIs) describe injuries sustained by the teeth, periodontium and soft tissues resulting from trauma.^1^ TDIs account for 5% of all injuries for which people seek treatment.^2^ TDIs can occur in both permanent and primary dentition, however, young children are at a greater risk of suffering from TDIs due to their undeveloped motor skills, with sports‐related injuries and altercations being a common cause. It can range from a simple enamel chip to an extensive maxillofacial trauma, with luxation injuries being the most common TDI in children.^3^ It is an oral health concern as TDIs can affect patients functionally, psychologically and socially.^4^

Several predisposing factors can lead to a higher incidence of TDIs, including increased overjet and insufficient lip cover, protrusion of upper incisors, anterior open bite, hyperactivity, poor motor coordination and epilepsy. A study showed that children with insufficient lip coverage are 1.95 times more prone to TDIs than those with sufficient lip coverage due to insufficient protections provided by the lip.^1^ On the other hand, it was noted that children with an anterior open bite had twice the level of TDIs compared to those with a normal occlusal relationship.^1^

Trauma of primary teeth can result in pain and affect the development of the permanent dentition.^5^ This is due to the close relationship between the apex of the injured primary tooth root and the underlying permanent tooth germ. Depending on the type of injury, occurrence of developmental defects in permanent successors ranges from 69% to 27%.^6^ Common effects on the permanent successors are enamel hypoplasia, crown dilaceration, root malformation and odontoma‐like teeth, with enamel hypoplasia being the most common.^6^

Such consequences indicate the importance of optimal management of TDIs as it can lower the incidence of the above‐mentioned developmental defects and increase the survival rate of the tooth. A study done by Ravin in 1981 showed that the rate of pulp necrosis significantly decreases with appropriate treatment from 54% pulpal necrosis when no treatment is provided to only 8% when appropriate treatment is provided.^7^ With that being said, the success of TDI treatment is multifactorial. Factors that can influence treatment include the child’s maturity and ability to cope with the emergency, the time for exfoliation of the injured tooth and the occlusion. In addition to the above factors, other compounding factors include poorer treatment outcomes in treating and examining children due to their lack of cooperation and fear of dental work, which they deem to be foreign.^8^

Management of TDIs would be slightly different in older children due to the presence of the permanent dentition. For example, the management of avulsion differs for primary or permanent teeth. An avulsed primary tooth should never be replanted. In comparison, an avulsed permanent tooth requires replantation. However, the prognosis of the avulsed permanent tooth is dependent on actions taken at the time of the incident. Replanting the tooth within a time period of 60 min or less is crucial to ensure periodontal ligament (PDL) cells are viable. Anything beyond this time frame can affect the vitality of the PDL cells and the maturity of the root, both of which play an important role in the revascularization of the tooth.^9^ The permanent dentition has great healing abilities even after traumatic pulp exposure, luxation injury and root fractures. This is especially so if it is an immature permanent tooth. Therefore, efforts should be made to preserve it to ensure continuous root development. This is because the loss of a tooth has a lifetime consequential effect on the child.

However, research has shown that parents and sports coaches have not received sufficient education regarding TDI management. One study found that only 5% of children with a TDI received treatment.^5^ Furthermore, just 4.1% of the children received dental care within the first 24 h following the incident, while 0.8% received dental care after 36 h.^5^ This shows that adults are not equipped with sufficient knowledge regarding the management of TDIs. Moreover, another study reported that only 7.3% of TDI cases were seen by Dentists, with the rest seen by Emergency Medical Technicians.^10^ This further emphasizes the lack of knowledge regarding the management of TDIs among primary caregivers for children. The majority of TDIs have been found to occur in homes, schools and streets, with the leading cause of injuries being falls and sporting injuries.^11^ Based on the conclusions of the study, it is highly likely that sports coaches and parents are present when the TDI occurs. Hence, as the first responders to the injuries, it is paramount for parents and coaches to understand the importance of appropriate management and act accordingly when faced with such situations.^12^

The same study concluded that greater emphasis has to be placed on educating primary caregivers on diagnosing and treating dental trauma.^10^ It was found that only 5.9% of medical practitioners received education on dental trauma management, leading to the conclusion that the average parent or coach with no medical background would have equal or less education on TDI management. Parents and coaches are likely to be the primary caregivers of their children when their children engage in high‐risk activities. Hence, the urgency for parents and coaches to be educated on the appropriate diagnosis and management of TDI.

Sports coaches are required by law to have first aid certificates. However, first aid courses do not include components regarding dental trauma. Since it is not a requirement for sports coaches to have basic knowledge in dental trauma, many coaches lack training in this aspect despite it being a common injury during sports school.^11^ With the knowledge of dental trauma, coaches can provide timely and appropriate measures, being the first to manage the injuries. This will significantly improve the prognosis of the traumatized tooth.

Education on TDI management should also include follow‐ups and home care as these help to improve healing of a TDI occurrence.^12^ Parents should be educated about the home care advice for optimal healing such as meticulous oral hygiene, rinsing with Chlorhexidine Gluconate 0.1% alcohol‐free for 1–2 weeks and avoiding contact sports to prevent further injury.^13^ In younger children, pacifier usage should be restricted, and a cotton swab can be used to apply Chlorhexidine Gluconate 0.1% on the affected area.^13^

Despite there being a strong correlation between appropriate early management and the better outcomes for traumatized teeth in children, the literature has demonstrated that parental and training coaches’ knowledge of TDI management is insufficient. Thus, the aim of this study is to investigate parents' knowledge regarding TDI and its management; and to identify effective strategies to rectify gaps in TDI knowledge.

## MATERIALS AND METHODS

This cross sectional study involved the recruitment of parents and training coaches to complete a 31‐item questionnaire. This questionnaire was designed to investigate the knowledge and attitude towards dental trauma management in children. The study was approved by the Human Research Ethics committee (HREC) of The University of Queensland (Approval number 2020000880). Gatekeeper approval was also obtained from sporting events such as soccer, netball, hockey, softball in Greater Brisbane.

### Study participants

Participants were recruited from the parents of children participating at different sporting clubs across Brisbane, accounting for 60 different suburbs. The inclusion criteria were parents whose children attended sport clubs in Brisbane, who agreed to participate in the study. The questionnaires were distributed physically via printed paper. The participant information sheet and consent form were distributed to every participant before starting the questionnaire. The data collection was performed from November 2020 to January 2021.

### Survey tool

A questionnaire was developed to assess participants’ self‐reported knowledge, attitudes and responses towards dental trauma in children under their care. The questionnaire was first piloted among a small group of individuals in the dental field (including students, dentists and paediatric specialists) before implementation, and modifications were made according to the feedback received. The questionnaire consisted of five categories. Category one contained six items that recorded demographics, educational background and information regarding previous dental trauma incidents experienced by their children. Category two had six items relating to assessing the participants’ knowledge, responses and attitudes towards different types of primary teeth traumatic injuries. Category three contained nine items that assessed participants regarding subluxation injuries and fractures of permanent teeth in children. Category four contained eight items that assessed participants’ knowledge regarding the avulsion of permanent teeth in children. The responses of categories two, three and four were assigned a score of 0 (incorrect) and 1 (correct) and were summed to provide an overall knowledge score for each participant. The possible knowledge score range was 0–21. The scores were converted to a percentage and classified into three levels as follows: a score of <50 was classified as a low level of knowledge, a score of 75 or more was classified as high level of knowledge, and the remaining scores was classified as moderate level of knowledge. The final category of the questionnaire contained seven items related to the overall need for continuing education for managing dental trauma in children. Most questions were in multiple‐choice format, with some questions including options of ‘other’ where the participants could elaborate further in text free fields if they deemed appropriate. All questions also had the option of selecting multiple options. The estimated completion time for the questionnaire was 10 min.

### Statistical analysis

The data were tabulated on a Microsoft Excel spreadsheet and then imported into IBM SPSS Statistics for Macintosh v26 (IBM, Armonk, NY, USA) for descriptive analysis and GraphPad PRISM 8.0 software (GraphPad Software, San Diego, CA, USA) for collation and creation of appropriate graphs. Responses were summarized, and comparisons were made. The output data were presented in a table format (total responses and percentage) and in graphical format. Specific data analysis tests that were performed included descriptive statistics, such as frequencies and percentages. The levels of statistical significance were determined using a chi‐square test, and a *P* ≤ 0.05 was considered statistically significant.

## RESULTS

### Demographics and information regarding the survey

A total of 233 (211 parents and 22 coaches) participants from approximately 50 sporting clubs completed the survey. Sporting clubs located in different areas of greater Brisbane (north, south, east and west) were chosen. This was to ensure that the results obtained are representative of the Greater Brisbane population. Brisbane is the capital of Queensland with a population of 2.5 million, the third most populated in Australia. The distribution of the participants relating to gender, education level and occupation status are shown in Table 1.

**Table 1:** Relationship of participants’ self‐assessment of their current knowledge and their need for future education stratified according to their demographic factors

Variable	n (%)	Knowledge		Need future education	
		Sufficient n (%)	Insufficient n (%)	Yes n (%)	No n (%)
Gender					
Male	54 (25.6)	15 (27.8)	39 (72.2)	14 (25.9)	40 (74.1)
Female	120 (56.9)	30 (26.1)	85 (73.9)	39 (33.3)	78 (66.7)
Education					
High school and below	27 (12.8)	9 (34.6)	17 (65.4)	6 (22.2)	21 (77.8)
Diploma or equivalent	24 (11.4)	2 (8.3)	22 (91.7)	2 (8.3)	22 (91.7)
Bachelor, masters	149 (70.6)	39 (26.9)	106 (73.1)	49 (33.8)	96 (66.2)
Occupation					
Educational	17 (8.0)	6 (35.3)	11 (64.7)	5 (29.4)	12 (71.6)
Health care	31 (14.6)	14 (48.3)	15 (51.7)	7 (31.8)	22 (68.2)
Administration/office work	98 (46.2)	14 (14.7)	81 (85.3)	34 (35.4)	62 (64.6)
Trades worker	23 (10.8)	7 (30.4)	16 (69.6)	6 (26.1)	17 (73.9)
Others	42 (19.8)	13 (31.0)	29 (69.0)	9 (21.4)	33 (78.6)

### Assessment of knowledge about management of TDIs

Of all types of injuries, parental knowledge of managing avulsion to the permanent teeth was poorest (9.5%), followed by management of primary tooth (17.5%) and management of fractures and subluxation of permanent tooth (29.4%). The distribution of knowledge levels by the satisfaction of current knowledge are shown in Tables 2 and 3.

**Table 2:** Distribution of responses segregated based on participant’s current dental trauma knowledge

Statements	Parental knowledge n (%)			Coaches knowledge n (%)		
	Yes	No	Other	Yes	No	Other
Management of primary TDI						
Seeking professional help when child is not in pain	138 (65.4)	60 (28.4)	13 (6.2)	13 (59.1)	7 (31.8)	2 (9.1)
Seeking dental help when tooth is wobbly, displaced or broken	145 (68.7)	33 (15.6)	33 (15.6)	14 (63.7)	3 (13.6)	5 (22.7)
Seeking dental help when tooth is knocked out	123 (58.3)	61 (28.9)	27 (12.8)	13 (59.1)	5 (22.7)	4 (18.2)
Placing baby tooth back into socket when knocked out	10 (4.7)	107 (50.7)	94 (44.5)	1 (4.6)	16 (72.7)	5 (22.7)
Management of fracture and subluxation to permanent teeth						
Seeking professional help immediately when child is in pain following injury	178 (84.4)	16 (7.6)	17 (8.1)	21 (95.4)	0 (0.0)	1 (4.6)
Seeking professional help immediately when child is bleeding from mouth following injury	101 (47.9)	94 (44.5)	16 (7.6)	20 (90.9)	1 (4.6)	1 (4.6)
Do you think it is important to find the missing fragment of a chipped tooth	71 (33.6)	120 (56.9)	20 (9.5)	18 (81.8)	3 (13.6)	1 (4.6)
Management of avulsion to permanent teeth						
Ever experienced an accident where a tooth was knocked out	50 (23.7)	141 (66.8)	20 (9.5)	11 (50.0)	8 (36.4)	3 (13.6)
If the tooth is knocked out, will you immediately put it back in the socket	73 (34.6)	116 (55.0)	22 (10.4)	6 (27.3)	16 (72.7)	0 (0.0)
Information and knowledge related to the management of TDI						
Satisfied with their knowledge on the management of dental trauma	54 (25.6)	103 (48.8)	54 (25.6)	7 (31.8)	8 (36.4)	7 (31.8)
Received any information on the management of traumatized teeth	32 (15.2)	173 (82.0)	6 (2.8)	3 (13.6)	17 (77.3)	2 (9.1)
Is it important to have an educational program in TDI management?	170 (80.6)	8 (3.8)	33 (15.7)	17 (77.3)	0 (0.0)	5 (22.7)
Would you like to attend an educational program on TDI management?	61 (28.9)	85 (40.4)	65 (30.7)	7 (31.8)	5 (22.7)	10 (45.5)

TDI = traumatic dental injury.

**Table 3:** Distribution of correct responses segregated based on parental and coaches knowledge.

Statements	Parental knowledge n (%)	Coaches knowledge n (%)
Management of primary TDI		
If the child is in pain after the injury, where would you seek professional help?		
Correct answer	139 (65.9)	9 (40.9)
Incorrect answer	72 (34.1)	13 (59.1)
What would you do if the soft tissue of the mouth (e.g. lips, gums, frenum, tongue) is cut?		
Correct answer	39 (18.5)	7 (31.8)
Incorrect answer	172 (81.5)	15 (68.2)
Management of fracture and subluxation to permanent teeth		
If the child is in pain after the injury, where would you seek professional help?		
Correct answer	70 (33.2)	4 (18.2)
Incorrect answer	141 (66.8)	18 (81.8)
What would you do if the soft tissue of the mouth (e.g. lips, gums, frenum, tongue) is cut?		
Correct answer	29 (13.7)	7 (31.8)
Incorrect answer	182 (86.3)	15 (68.2)
What would you do if the tooth is wobbly (mobile, shaky)?		
Correct answer	111 (52.6)	12 (54.6)
Incorrect answer	100 (47.4)	10 (45.4)
What would you do if the tooth is displaced (not in its usual position)?		
Correct answer	164 (77.7)	17 (77.3)
Incorrect answer	47 (22.3)	5 (22.7)
What would you do if the tooth is chipped (broken)?		
Correct answer	183 (86.7)	19 (86.4)
Incorrect answer	28 (13.3)	3 (13.6)
Management of avulsion to permanent teeth		
How urgent do you think it is to seek professional help if a permanent tooth has been knocked out?		
Correct answer	125 (59.2)	14 (63.6)
Incorrect answer	86 (40.8)	8 (36.4)
If you decide to put the tooth back into the socket but it had fallen onto the ground and covered with dirt, what would you do?		
Correct answer	100 (47.4)	13 (59.1)
Incorrect answer	111 (52.6)	9 (40.9)
If you wash the tooth what would you use to wash it?		
Correct answer	10 (16.4)	17 (81.0)
Incorrect answer	51 (83.6)	4 (19.0)
If you did not put the tooth back into the socket, how would you transport it to the dentist?		
Correct answer	38 (18.0)	4 (18.2)
Incorrect answer	173 (82.0)	18 (81.8)
If you used a liquid to transport the tooth to the dentist, what liquid would you use?		
Correct answer	67 (31.7)	8 (36.4)
Incorrect answer	144 (68.3)	14 (63.6)

TDI = traumatic dental injury.

Scores were tabulated from the responses collected, and the classification (stated in the survey tool) was used to categorize the level of knowledge of both the parents and coaches. The majority of participants fell under the category of moderate level of knowledge (between 50–75%), with 58.1% of parents and 59.1% of coaches in this category. Only 8.1% of parents and 13.6% of coaches displayed a high level of knowledge (75–100%). Coaches had a higher mean score for level of knowledge than parents, with the mean difference in scores being 3.95. The average score of coaches was 56.71 while parents were 52.76. Independent *t*‐test showed no significant differences in the level of knowledge between coaches and parents (*P* = 0.305).

### Assessment of attitudes regarding TDI management

The responses to statements exploring the importance placed on prioritizing treatment of TDI in children are presented in Table 4. The results showed that 72.8% of parents and 43.5% of coaches recognize the need of seeking help from the dentist when a TDI occurs to the primary dentition. However, only 21.3% of the parents and 33.3% of the coaches will seek dental help for soft tissue injury. 18.5% of the parents will self‐treat if their child experiences pain after a TDI of permanent teeth. About one in three parents (36.7%) and coaches (35.0%) would wait until a permanent tooth is wobbly after the dental trauma before seeking help. More than 85% of both parents’ coaches will seek professional help if the tooth was displaced and chipped. Over 65% of coaches and parents agreed that professional help would be sought immediately when a permanent tooth is avulsed.

**Table 4:** Distribution of actions taken by participants in the event of TDI

Statements	Parental knowledge n (%)	Coaches knowledge n (%)
Management of primary TDI		
If the child is in pain after the injury, where would you seek professional help?		
General medical practitioner	29 (11.9)	3 (13.0)
Accident and emergency department of hospital	37 (15.3)	10 (43.5)
General dental practitioner/specialist dentist	172 (71.1)	10 (43.5)
School dentist/therapist (e.g. dental vans)	4 (1.7)	0 (0.0)
Other	0 (0.0)	0 (0.0)
What would you do if the soft tissue of the mouth (e.g. lips, gums, frenum, tongue) is cut?		
Watch and wait	92 (28.4)	3 (11.1)
Clean the wound	83 (25.6)	5 (18.5)
Stop bleeding	76 (23.5)	10 (37.0)
Seek professional help (dentist/dental specialist)	69 (21.3)	9 (33.3)
Other	4 (1.2)	0 (0.0)
Management of fracture and subluxation to permanent teeth		
If the child is in pain after the injury, where would you seek professional help?		
Self‐treat	52 (18.5)	2 (6.5)
General medical practitioner	59 (21.0)	4 (12.9)
Accident and emergency department of hospital	45 (16.0)	13 (41.9)
General dental practitioner/specialist dentist	121 (43.1)	11 (35.5)
School dentist/therapist (e.g. dental vans)	4 (1.4)	1 (3.2)
Other	0 (0.0)	0 (0.0)
What would you do if the soft tissue of the mouth (e.g. lips, gums, frenum, tongue) is cut?		
Watch and wait	98 (30.2)	1 (3.0)
Clean the wound	85 (26.2)	8 (24.2)
Stop bleeding	76 (23.4)	12 (36.4)
Seek professional help (dentist/dental specialist)	64 (19.7)	12 (36.4)
Other	2 (0.6)	0 (0.0)
What would you do if the tooth is wobbly (mobile, shaky)?		
Watch and wait	77 (36.7)	7 (35.0)
Seek professional help (dentist/dental specialist)	121 (57.6)	13 (65.0)
Remove the tooth	3 (1.4)	0 (0.0)
Reposition the tooth	8 (3.8)	0 (0.0)
Other	1 (0.5)	0 (0.0)
What would you do if the tooth is displaced (not in its usual position)?		
Watch and wait	22 (10.7)	2 (10.5)
Seek professional help (dentist/dental specialist)	174 (84.9)	17 (89.5)
Remove the tooth	2 (1.0)	0 (0.0)
Reposition the tooth	6 (2.9)	0 (0.0)
Other	1 (0.5)	0 (0.0)
What would you do if the tooth is chipped (broken)?		
Watch and wait	10 (5.0)	0 (0.0)
Seek professional help (dentist/dental specialist)	187 (93.5)	19 (100.0)
Remove the tooth	0 (0.0)	0 (0.0)
Reposition the tooth (attempt to place the fragment back)	2 (1.0)	0 (0.0)
Other	1 (0.5)	0 (0.0)
Management of avulsion to permanent teeth		
How urgent do you think it is to seek professional help if a permanent tooth has been knocked out?		
Immediately	128 (65.3)	15 (65.2)
Within 30 min	15 (7.7)	2 (8.7)
Within a few hours	33 (16.8)	4 (17.4)
Before the next day	15 (7.7)	2 (8.7)
Not required	3 (1.5)	0 (0.0)
Other	2 (1.0)	0 (0.0)
If you decide to put the tooth back into the socket but it had fallen onto the ground and covered with dirt, what would you do?		
Remove the dirt gently with toothbrush	26 (13.4)	3 (12.5)
Rinse the tooth under tap water	111 (57.2)	15 (62.5)
Put the tooth back into the socket without doing anything	3 (1.5)	0 (0.0)
Don’t know	37 (19.1)	3 (12.5)
Other	17 (8.8)	3 (12.5)
If you wash the tooth what would you use to wash it?		
Tap water	102 (42.1)	12 (42.9)
Fresh milk	45 (18.6)	1 (3.6)
Fruit juice	30 (12.4)	0 (0.0)
Alcohol	8 (3.3)	1 (3.6)
Normal saline solution	33 (13.6)	9 (32.1)
Iced water	4 (1.7)	0 (0.0)
Antiseptic solution	17 (7.0)	5 (17.9)
Other	3 (1.2)	0 (0.0)
If you did not put the tooth back into the socket, how would you transport it to the dentist?		
In ice	22 (11.2)	5 (20.0)
In a liquid	41 (20.8)	4 (16.0)
In the child’s mouth	13 (6.6)	0 (0.0)
In a hand	4 (2.0)	0 (0.0)
In a tissue or clean handkerchief	76 (38.6)	11 (44.0)
In plastic wrap	36 (18.3)	5 (20.0)
Other	5 (2.5)	0 (0.0)
If you used a liquid to transport the tooth to the dentist, what liquid would you use?		
Tap water	80 (43.2)	8 (36.4)
Fresh milk	46 (24.9)	2 (9.1)
Fruit juice	0 (0.0)	0 (0.0)
Alcohol	4 (2.2)	0 (0.0)
Normal saline solution	30 (16.2)	7 (31.8)
Iced water	12 (6.5)	3 (13.6)
Antiseptic solution	9 (4.9)	2 (9.1)
Other	4 (2.2)	0 (0.0)

TDI = traumatic dental injury.

### Information and knowledge related to the management of TDIs

**Fig. 1:**
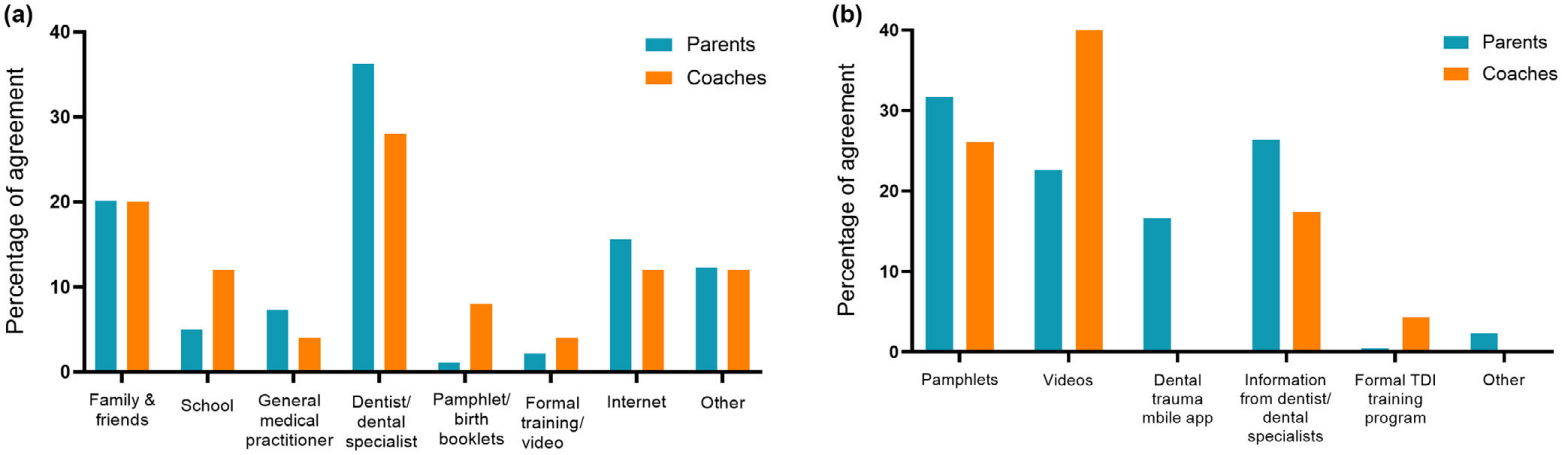
Information and knowledge related to the management of TDI (a) Responses for the statement: Where have you received advice on the management of traumatized teeth? (b) Responses for the statement: What sort of dental management program would you like to attend?

The results showed that only 26.2% of parents and 31.8% of coaches were satisfied with their current level of knowledge. Most parents (80.6%) and coaches (77.3%) acknowledge the importance of an educational program in TDI management. However, only 29.6% of parents and 31.8% were interested in attending such programs. The online platform is the most preferred medium, by both parents (62.1%) and coaches (57.1%) to access TDI management knowledge. The results of the distribution of parents and coaches preferred platforms to receive TDI information are given in Fig. 1. It was also observed that parents in health care occupations had higher satisfaction on self‐knowledge in managing a TDI however there was no significant difference in knowledge levels between health care personnel and other professions (*P* = 0.128).

## DISCUSSION

This study provides insight into TDI management knowledge among parents and coaches in various sporting clubs in Greater Brisbane, Australia. The study showed that knowledge level was most unsatisfactory for the avulsion of permanent teeth, followed by management of primary teeth and management of fractures and subluxation of permanent teeth. Previous research has showed a correlation between a high level of parental/coach knowledge to an improved prognosis of the injured tooth. The present study suggests the need for additional educational programs targeting coaches and parents to minimize and manage such injuries more effectively.

The present study showed that most parents and coaches would seek professional help following a TDI, indicating their recognition of the severity of the injury. However, in most cases, their primary contact for the management of a TDI was a medical practitioner instead of the dentist. These results are in line with the study done by Bazina *et al*. that showed only 20% of coaches would seek dental help after a TDI compared to 49% who preferred to visit a medical practitioner.^14^ In contrast with the above studies, a study by Quaranta *et al*. showed that almost 85% of the participants preferred to seek dental help following a TDI.^15^ In view of this, it might be beneficial to explore whether parents can identify signs of TDIs as this might influence their decision to seek dental help. Medical practitioners might not be trained, confident or well equipped for managing dental trauma. Parents and coaches should understand limitations of medical practitioners in managing these cases and should be aware of the correct dental pathways to follow in case of an emergency.

The most significant gap in knowledge of a TDI was in the management of avulsed permanent teeth in this study. Time plays a critical role in determining the prognosis and successful recovery of avulsed permanent teeth.^8^ The viability of the PDL cells is dependent on the time out of the mouth and on the storage medium in which the avulsed tooth is kept. Thus, minimizing dry time is critical for survival of PDL cells.^8^ After an extra‐alveolar dry time of 30 min, most PDL cells are non‐viable. If the tooth has been kept in an appropriate storage medium and the total extra‐oral dry time has been <60 min than the PDL cells might be viable but compromised.^8^ Anything beyond this time period, the PDL cells are likely to be non‐viable and has a poor long‐term prognosis.^8^ However, the study found that only a limited number of parents (35%) and coaches (27%) were aware of immediately replanting the tooth into the sockets. Common reasons cited by parents and coaches for not replanting teeth include being unaware of the option to replant teeth, the teeth are dirty and the inability to replant due to the child being distressed following trauma. The results imply a gap in both parental and coach knowledge in managing avulsed permanent teeth. In addition, only 30% of respondents correctly identified a suitable transport medium for avulsed permanent teeth. The transport medium of the avulsed tooth plays a significant role in determining the prognosis of that tooth. Multiple studies have shown that milk and Hanks' Balanced Salt Solution used as a transport medium of the avulsed tooth, could greatly improve its prognosis.^16^, ^17^, ^18^ The results of this study are in line with other studies showing similar results where 2–24.3% of the respondents choose the correct transport medium.^14^, ^15^, ^19^, ^20^ The finding of this study showed significant gaps in knowledge regarding the management of the avulsed permanent teeth and its transportation, thus targeted TDI educational programs are required to increase the awareness.

Seeking early professional help following TDIs of primary teeth could reduce the impact of the trauma on the permanent dentition. Depending on the type of injury, it might be possible to identify the complications that might arise. Thus, it can be monitored or addressed accordingly. This study found that most parents (65%) and coaches (59.1%) were willing to seek professional help after an injury to primary teeth even if the child is not in pain. The results of this study are in line with studies done in Kolkata where 84.6% of parents would still seek professional help in the absence of any pain after a primary tooth TDI.^19^ This shows that the parental inability to identify TDI likely accounts for instances where parents do not seek professional help after such an incident. This further reinforces the importance of educating parents about TDIs to identify its signs and symptoms and seek appropriate professional assistance.

TDIs are one of the most common injuries sustained during sports. Parents and coaches play the role of the first responders in a majority of these cases. Without sufficient knowledge, they are unable to manage TDIs. This study found no significant difference between the parental and coaches knowledge levels in TDI management. Furthermore, the majority of the coaches did not feel confident in managing these injuries. This corresponds with other studies investigating the knowledge of TDI management of water polo and taekwondo sports coaches.^14^, ^21^ Although participants did recognize the importance of receiving information on TDI management, however, only 30% of participants were willing to attend TDI educational programs. This is in contrast with an Indian study where over 80% of the participants were willing to attend such a program.^19^ In was also found in this study that regardless of gender (25.9–33.3%), level of education (22.2–33.8%) or occupation (21.4–35.4%), that there was a lack of correlation for their desire to increase their knowledge on the management of TDIs (Table 1). It is suspected that there is an underlying parental attitude as well as a missed opportunity in early childhood caregiving. There is a need for the implementation of various approaches to enhance the awareness and motivations of both parents and coaches to receive information related to TDIs. Multiples strategies could be utilized such as incentives and posters regarding TDI management. It should be distributed to various sporting clubs and TDI management should be a mandatory section of the first aid course nationwide.

The International Association of Dental Traumatology (IADT) has played a significant role in developing guidelines to manage the incidence of TDIs. The latest guidelines to manage TDIs has been updated in 2020, which can be found on the IADT official website.^8^, ^12^, ^22^ Following these guidelines during TDI management is crucial to ensure the best prognosis of the injured teeth. This has been proven by Bücher *et al*. in which they found significantly lower complications and higher survival rate of teeth when they are managed in accordance with IADT guidelines as compared to cases that were managed without the guidelines.^23^ Therefore, to increase parents’ and coaches’ knowledge regarding TDI, awareness regarding mobile applications like ‘IADT ToothSOS’ and the dental trauma guideline (DTG) should be raised. ToothSOS is an application launched by the IADT. This application aims to provide information to manage TDI for both the general public and professionals. Studies have shown that this application has had 47 725 downloads over the 2‐years since it was launched in 2018.^24^ Users come from various territories such as Europe, USA, Asia, Canada and many more. In another study done, they found that younger dentists had significantly higher dental trauma knowledge compared to older dentists.^25^ The findings might be attributed to the fact that younger generations are generally more proficient with modern technology and hence have easier access to websites or ToothSOS applications to find out more information regarding DTGs. Another study concluded that dental students who used the application arrived at the same diagnosis as their experienced dental supervisors.^26^ Hence, such studies show that the application and guidelines are accurate and effective in providing TDI management information. Hence, such platforms will be greatly beneficial for both parents and coaches.

Managing TDIs is complicated due to the numerous possible nature and types of injuries resulting from trauma. A study carried out in Denmark concluded that there are more than 100 TDI scenarios, with each scenario requiring an accurate diagnosis and specific treatment.^27^ An online guide provides a systematic approach in diagnosing and managing TDI. A recent study carried out in Brazil found dentists to have a moderate level of knowledge regarding TDI management when assessed with the IADT Guidelines.^28^ These studies highlighted the need for additional education regarding TDI management. Improvements in TDI management knowledge have been demonstrated with the use of the DTG. Another study carried out in Sri Lanka found that dental students with access to the DTG website had a higher TDI knowledge score than students without access.^29^ Hence, websites such as the DTG are valuable platforms to help users arrive at an evidence‐based diagnosis. An accurate diagnosis would aid in appropriate management of the TDI. At the end of this survey, parents and coaches were given TDI information in the form of pamphlets, introduced to the ToothSOS application and informed of the dental trauma guide website. All these efforts were in the hope to increase their knowledge regarding TDI management in future.

This study was undertaken during the Covid‐19 pandemic, when there were multiple on and off restrictions in place. Thus, the sample size was a convenience sample of the active sporting clubs during that time. However, future nationwide studies with a larger sample sizes, including various sports, are recommended to draw more representative results for coaches. In addition, future studies should evaluate the use of mouth‐guards during sports as this study did not collect data regarding awareness and usage of mouth‐guard in children. Previous studies have shown the effectiveness of mouth‐guards in preventing and reducing the rate of TDIs during sport.^30^, ^31^, ^32^ As there is no previous study done in Brisbane, no comparison could be made regarding how the knowledge level has changed over the years. Future similar studies should be carried out in different parts of Australia so a national strategy could increase awareness and knowledge on TDI management in Australia.

## CONCLUSION

The study showed a gap in parents’ and training coaches’ knowledge regarding TDI management in children, especially with avulsion cases. However, there was no significant difference in the level of knowledge between parents and coaches. It was also observed that parents and coaches acknowledged shortcomings in their dental trauma knowledge, however, lacked willingness to further self‐educate. However, parents and coaches were receptive to accessible TDI management educational programs, especially via online platforms. These findings might be useful when designing future TDI management educational programs.

## CONFLICT OF INTEREST

The authors have no conflict of interest to declare.
